# Opposing Effects of α2- and β-Adrenergic Receptor Stimulation on Quiescent Neural Precursor Cell Activity and Adult Hippocampal Neurogenesis

**DOI:** 10.1371/journal.pone.0098736

**Published:** 2014-06-12

**Authors:** Dhanisha J. Jhaveri, Ishira Nanavaty, Boris W. Prosper, Swanand Marathe, Basma F. A. Husain, Steven G. Kernie, Perry F. Bartlett, Vidita A. Vaidya

**Affiliations:** 1 Queensland Brain Institute, The University of Queensland, Brisbane, Queensland, Australia; 2 Department of Biological Sciences, Tata Institute of Fundamental Research, Mumbai, India; 3 Departments of Pediatrics and Pathology and Cell Biology, Columbia University College of Physicians and Surgeons, New York, New York, United States of America; Robert Debre Hospital, France

## Abstract

Norepinephrine regulates latent neural stem cell activity and adult hippocampal neurogenesis, and has an important role in modulating hippocampal functions such as learning, memory and mood. Adult hippocampal neurogenesis is a multi-stage process, spanning from the activation and proliferation of hippocampal stem cells, to their differentiation into neurons. However, the stage-specific effects of noradrenergic receptors in regulating adult hippocampal neurogenesis remain poorly understood. In this study, we used transgenic Nestin-GFP mice and neurosphere assays to show that modulation of α2- and β-adrenergic receptor activity directly affects Nestin-GFP/GFAP-positive precursor cell population albeit in an opposing fashion. While selective stimulation of α2-adrenergic receptors decreases precursor cell activation, proliferation and immature neuron number, stimulation of β-adrenergic receptors activates the quiescent precursor pool and enhances their proliferation in the adult hippocampus. Furthermore, our data indicate no major role for α1-adrenergic receptors, as we did not observe any change in either the activation and proliferation of hippocampal precursors following selective stimulation or blockade of α1-adrenergic receptors. Taken together, our data suggest that under physiological as well as under conditions that lead to enhanced norepinephrine release, the balance between α2- and β-adrenergic receptor activity regulates precursor cell activity and hippocampal neurogenesis.

## Introduction

The mammalian hippocampal neurogenic niche retains quiescent neural precursor cells that generate newborn neurons throughout life [1], [2]. This process of adult hippocampal neurogenesis is a unique form of structural plasticity that has been implicated in the regulation of hippocampus-specific cognitive and mood-related functions [3], [4]. Though we know the process is tightly controlled and subject to regulation at various stages, including the activation and proliferation of precursors, as well as their differentiation, survival and integration into existing functional networks [3], [5], [6], the detailed molecular mechanisms that regulate of each of these stages are not yet fully elucidated.

The neurogenic niche in the adult hippocampus is densely innervated by monoaminergic axon terminals, particularly noradrenergic terminals that arise from locus coeruleus neurons in the brain stem [7]. Numerous studies have shown norepinephrine to have a positive effect on hippocampal neurogenesis [1], [8], [9] and the modulation of neurogenesis-related functions such as learning, memory and mood [10]–[13]. Our previous work has demonstrated that pharmacological depletion of norepinephrine leads to a robust decline in hippocampal precursor cell proliferation [8] and, more recently, we have shown that norepinephrine directly activates a quiescent population of hippocampal stem/precursor cells [1]. Interestingly, clinical antidepressants that block the re-uptake of norepinephrine have also been reported to increase precursor cell proliferation and enhance hippocampal neurogenesis [1], [9].

Norepinephrine signals via a family of adrenergic receptors comprised of three major classes, α1-, α2- and β-adrenergic receptors, which are coupled to distinct intracellular signalling pathways [14]. We have previously shown that stimulation of α2-adrenergic receptors inhibits and β3-adrenergic receptor stimulation activates hippocampal precursor activity both *in vitro* and *in vivo* [1], [15]. Recent reports also suggest enhanced hippocampal progenitor turnover in response to β2-adrenergic receptor stimulation [16]. Although these studies suggest differing effects of these specific adrenergic receptors on hippocampal neurogenesis, we do not yet have a clear understanding of the stage-specific effects of adrenergic receptor activity in the regulation of hippocampal neurogenesis.

In the present study, we systematically investigated the effects of three major subclasses of adrenergic receptors in regulating specific stages of hippocampal neurogenesis. Using the neurosphere assay and a Nestin-GFP transgenic reporter mouse line, in which hippocampal precursor cells are labeled, we examined the effects of selective adrenergic receptor agonists and antagonists on hippocampal precursor cell activation, proliferation and differentiation. The term activation of hippocampal precursors indicates the acquired ability of latent, non-proliferating hippocampal precursor cells to respond to mitogens *in vitro* and generate neurospheres. Our findings indicate that stimulation of α2-adrenergic receptors significantly reduces the activation and proliferation of the quiescent precursor cells. In contrast and as previously reported, we found stimulation of β-adrenergic receptors activates these precursor cells and increases their proliferation. Moreover, blockade of β-adrenergic receptors leads to a significant decline in quiescent and active precursor cell populations and hippocampal neurogenesis. More importantly, we now show that while stimulation of α2- adrenergic receptors directly inhibits the Nestin-GFP-positive precursor cell population, treatment with β-adrenergic receptor agonist results in activation of this population. Furthermore, our results indicate no major role for the α1-adrenergic receptor in regulating adult hippocampal neurogenesis. These findings reveal that norepinephrine acts through the α2- and β-adrenergic receptors to exert a direct but opposing effect on quiescent neural precursor cell activity and hippocampal neurogenesis.

## Materials and Methods

### Animals

Adult (8–12-week-old) male C57BL/6J mice were used for all the *in vitro* experiments conducted in this study. 8–12 week-old transgenic Nestin-GFP mice [17] were used to address the stage-specific effects of adrenergic receptor manipulations on adult hippocampal precursor cells and to isolate and enrich for hippocampal precursor cells by flow cytometry. The Nestin-GFP mice were bred on a C57BL/6J background and express green fluorescent protein (GFP) under the control of the Nestin promoter, thereby allowing visualization of the endogenous population of neural precursors. All mice were housed in groups and maintained on a 12-hour light/dark cycle with *ad libitum* access to food and water.

### Ethics Statement

All animal treatments and experimental procedures were carried out in accordance with the National Institutes of Health Guide, the Australian Code of Practice for the Care and Use of Animals for Scientific Purposes and the guidelines of the Indian Committee for the Purpose of Care and Supervision of Experimental Animals. The experimental procedures were approved by the Tata Institute of Fundamental Research Institutional Animal Ethics Committee and The University of Queensland Animal Ethics Committee.

### Drugs and treatment

Selective agonists or antagonists of different adrenergic receptors were used to study receptor effects on adult hippocampal neural precursors and neurogenesis. All drugs were purchased from Sigma-Aldrich and the *in vitro* concentrations used and the *in vivo* dose administered are summarized in Table 1. For *in vivo* treatment, all drugs were administered intraperitoneally (i.p.) once daily for 7 days. The choice of drug dose was based on previous studies [18]–[23]. 5-bromo-2-deoxyuridine (BrdU; 100 mg/kg; Sigma), a thymidine analogue that is incorporated into the DNA during the S-phase of the cell cycle, was used to label proliferating precursors in the dentate gyrus. The animals were administered BrdU 2 h after the last drug injection and were sacrificed 24 h later (n = 4–6 mice per group). Each treatment had its own vehicle group with 10% DMSO used as a vehicle for yohimbine and prazosin, and 0.9% saline used for all other drugs.

**Table 1:** Dose and concentrations of sub-class-selective adrenergic receptor agonists and antagonists used in this study.

	Receptors	Drugs	*In vivo* (mg/kg/day)	*In vitro* (µM)
**AGONISTS**	α1-ARs	Cirazoline	0.5	0.1, 1 and 10
	α2-ARs	Guanabenz	2	0.1, 1 and 10
	β-ARs	Isoproterenol	2	0.1, 1 and 10
**ANTAGONISTS**	α1-ARs	Prazosin	1	0.01, 0.1 and 1
	α2-ARs	Yohimbine	2	0.1, 1 and 10
	β-ARs	Propranolol	1	0.1, 1 and 10

### Adult hippocampal neurosphere culture

Adult male C57BL/6J mice were killed by cervical dislocation and their brains were removed and placed in ice-cold Hank's essential medium. The hippocampi were isolated from the overlying cortex and minced using a scalpel blade. Minced tissue was digested in 0.1% papain (Invitrogen) for 16 min at 37°C, during which time the mixture was triturated gently to obtain a single cell suspension. An excess of NeuroCult NSC basal medium (Stem Cell Technologies) was added to halt the digestion, and the cell suspension was centrifuged at 100 rcf for 5 min. The resulting pellet was resuspended in 1 ml of complete neurosphere medium, i.e. NeuroCult NSC basal medium containing NeuroCult proliferation supplements (StemCell Technologies), 2% bovine serum albumin (Invitrogen) 2 µg/ml heparin (Sigma-Aldrich) and growth factors including 20 ng/ml epidermal growth factor (EGF; receptor grade, BD Biosciences) and 10 ng/ml basic fibroblast growth factor (bFGF; recombinant bovine, Roche). The cells were then plated in a 96-well plate and cultured in complete neurosphere medium containing EGF and bFGF in either the presence or absence of L-(−)-noradrenaline (+)-bitartrate salt monohydrate (norepinephrine; 10 µM). The dose responses of selective adrenergic receptor agonists and anatgonists were tested at various concentrations (detailed in Table 1). Average live cells obtained from 2 hippocampi were 3.54±0.08×10^5^ and half of these cells were used for each of the treatment conditions and plated in 48 wells. The medium containing growth factors was not replaced during the 14-day term of the culture. On day 14, a total count of the primary neurospheres was conducted for each of the treatments and neurospheres were categorized based on size. Values were expressed as a percentage relative to the control.

### Neurosphere differentiation and immunocytochemistry

Control and treated neurospheres were collected on day 14 and plated onto poly-D-lysine-coated BioCoat 8-well culture slides (BD Biosciences) in serum-free basal medium (without mitogens). The neurospheres were allowed to flatten and adhere for an additional 4 to 5 days in a humidified, 5% CO_2_ incubator. They were then fixed with ice-cold 4% paraformaldehyde in 0.1 M phosphate-buffered saline (PBS) for 30 min at 4°C and immunocytochemistry was performed as described previously (Jhaveri et al., 2010), using antibodies to the neuronal marker βIII tubulin (1∶2000; Promega), and the astrocytic marker glial fibrillary acidic protein (GFAP; 1∶500; Dako Cytomation). 4′,6′-diamidino-2-phenylindole (DAPI; 1∶5000; Sigma-Aldrich) was used as a nuclear stain. Slides were mounted using fluoromount (Dako Cytomation) and viewed on a Zeiss-Axio Imager microscope. Images were captured using a digital camera linked to a computer using Zeiss software.

### Fluorescence-activated cell sorting

Hippocampi from 3–4 Nestin-GFP mice (7–9 week old) were isolated and a live-cell suspension prepared, as described earlier. The dead cells were labeled with propidium iodide (1 µg/ml). GFP-positive and -negative cells were sorted by flow cytometry using FACS Aria (Becton Dickinson, Australia). The GFP-positive population was defined relative to the basal fluorescence levels obtained from wild-type controls. The GFP-positive cells were collected in neurosphere medium and plated into 96-well tissue culture plates in four different conditions: EGF+bFGF only (control), guanabenz (10 µM), isoproterenol (1 µM) or norepinephrine (10 µM). The number of neurospheres obtained in each of the conditions was quantified on day 14 and plotted as a percentage of the control.

### Immunohistochemistry

Animals were sacrificed by transcardial perfusion with ice-cold 4% paraformaldehyde (PFA), after which their brains were removed and post-fixed in PFA. Serial coronal sections (50 µm thick) through the rostro-caudal axis of the hippocampus were generated using a vibratome (Leica). For BrdU immunohistochemistry, every sixth section was selected and processed for quantification (eight sections per animal). Briefly, BrdU immunohistochemistry involved DNA denaturation and acid hydrolysis, followed by incubation with mouse anti-BrdU antibody (1∶250; Roche) overnight at 4°C. Sections were then incubated with secondary antibody (biotinylated anti-mouse IgG, 1∶500; Vector Laboratories) for 3 h before amplification with an avidin-biotin complex (Vector Laboratories) and visualization with diaminobenzidine (Sigma). Sections were mounted using DPX (Qualigen, India).

The number of immature neurons in the subgranular zone (SGZ) was evaluated by performing doublecortin (DCX) immunohistochemistry on four equivalent sections per animal for control and treated groups. The sections were incubated overnight with anti-DCX antibodies (goat anti-DCX, 1∶250, Santa Cruz Biotechnology or rabbit anti-DCX, 1∶500, Abcam) before being incubated with secondary antibodies, as described above.

The effect of adrenergic receptor perturbations on the Nestin-positive pool of adult hippocampal precursors was quantified using four sections per animal. Sections were incubated overnight with rabbit anti-GFP (1∶1000; Molecular Probes) followed by incubation in the secondary antibody (Alexa 488-conjugated donkey anti-rabbit, 1∶250; Molecular Probes) for 3 h. Double immunohistochemistry for GFP and GFAP was carried out to label the quiescent neural precursor population. The sections were incubated overnight at room temperature in a cocktail of the primary antibodies, rabbit anti-GFP (1∶500; Invitrogen) and mouse anti-GFAP (1∶1000; Sigma). Sections were washed using 0.1 M PBS and incubated for 3 h at room temperature with the secondary antibody, a mixture of donkey anti-rabbit Alexa-488 and donkey anti-mouse Alexa-547 (1∶250; Molecular Probes). Sections were mounted using Vectashield (Vector) and imaged using confocal microscopy.

### Cell counting analysis

The slides containing the sections were coded and quantification performed by an experimenter blind to the code. BrdU-positive cells were counted within the SGZ [15] on a Zeiss Axioskop-2 Plus microscope. The number of BrdU-positive cells per section was determined by assessing every sixth section across the rostro-caudal extent of the hippocampus (10 sections/animal). The number of DCX- and GFP-positive cells in the dentate gyrus was quantified (four sections per animal) using a Zeiss Axioscope and Nikon Eclipse 90i fluorescence microscope, respectively. The cell counts are plotted as a percentage of the control (vehicle-treated) for each adrenergic receptor agonist and antagonist. The numbers of cells/section for all treatment conditions are shown in Table S1.

The number of Nestin-GFP/GFAP double-positive cells was quantified by determining the percentage of GFP-positive cells that colocalized with GFAP using confocal microscopy. At least 50 GFP-positive cells from each animal (four sections per animal) were analyzed using z-plane confocal sectioning with 1 µm steps on a Zeiss LSM5 Exciter microscope.

### Statistical analysis

All data are expressed as mean ± standard error of the mean (SEM). Results were subjected to statistical analysis using the statistical software Prism (GraphPad) and analyzed using a Student's unpaired *t*-test for experiments with two groups or one-way ANOVA for experiments with three groups or more followed by the Bonferroni post hoc test. Significance was determined at *p*<0.05.

## Results

### α1-adrenergic receptors do not regulate hippocampal precursor activity or new neuron production in the adult hippocampus

**Figure 1:**
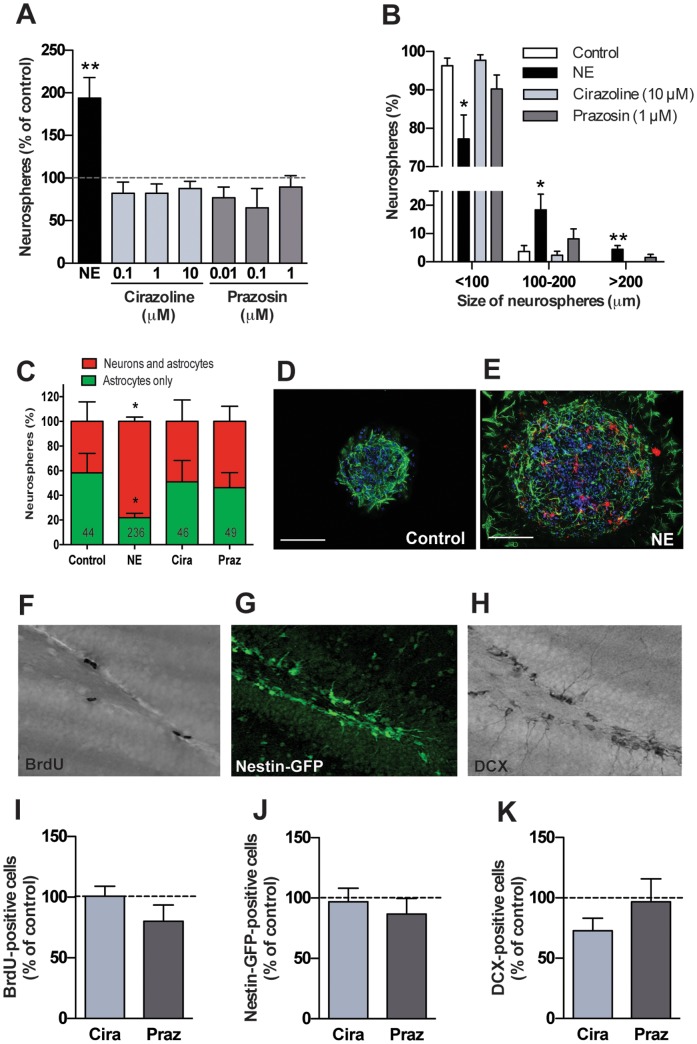
Stimulation or blockade of α1-adrenergic receptors does no affect hippocampal precursor activity or neurogenesis. (A) Treatment of adult hippocampal cells with norepinephrine (NE) leads to 2-fold increase in neurosphere numbers, however, treatment with cirazoline or prazosin does not affect neurosphere formation at any dose (n = 4 experiments). (B) Neurospheres obtained in presence of norepinephrine were significantly larger than that obtained in control medium containing EGF and bFGF. Again, cirazoline- or prazosin-treated neurospheres were comparable to control (n = 4 experiments). (C) Relative percentage of the primary neurospheres expressing markers of astrocytes and neurons in control, norepinephrine, cirazoline and prazosin-treated cultures (n = 5 experiments). While all neurospheres examined contained GFAP-positive astrocytes, a significantly larger proportion of neurospheres expressed the neuronal marker, βIII tubulin, in the norepinephrine-treated versus the control, cirazoline or prazosin group. The total number of neurospheres examined for each treatment group is indicated on the graph. An example of control (D) and NE-derived (E) neurospheres showing immunofluorescence for GFAP (green) and βIII tubulin (red). Nuclei were stained with DAPI (blue). Scale bars represent 100 µm. Note the presence of βIII tubulin-positive neurons in the norepinephrine-derived large neurosphere. Percentage of BrdU-labeled cells in the SGZ was not altered following treatment with either cirazoline or prazosin once daily for 7 days (n = 5-6 mice per group, F, I). Also, cirazoline or prazosin did not change the pool of Nestin-GFP-positive cells compared to the vehicle-treated control (G, J). Similarly, the number of DCX-positive immature neurons remained unaltered following stimulation or blockade of α1-adrenergic receptors (H, K). (mean±SEM, *p<0.05, **p<0.01).

α1-adrenergic receptors are Gq-coupled receptors that are widely expressed in the hippocampus [24], [25] and have been reported to stimulate proliferation in the subventricular zone and also in the SGZ [26]. To determine whether α1-adrenergic receptors play a role in regulating adult hippocampal neurogenesis, we first evaluated their effect on hippocampal precursor activity *in vitro* using the selective α1-adrenergic receptor agonist, cirazoline or the antagonist, prazosin. The neurosphere assay was performed on a single-cell suspension of adult hippocampal cells containing precursor that were treated with 100 nM, 1 µM, or 10 µM cirazoline or prazosin in the presence of EGF and bFGF. Treatment with 10 µM norepinephrine was used as a positive control based on our previous findings that showed norepinephrine-mediated activation of a quiescent population of adult hippocampal precursor cells [1]. In contrast to norepinephrine treatment, which resulted in a ∼2-fold increase in the number of neurospheres, treatment with cirazoline or prazosin did not change the total number of neurospheres obtained compared to the control (Fig. 1A). Moreover, whereas 10 µM norepinephrine resulted in a significant increase in the proportion of large neurospheres (>200 µm in diameter), reflective of activation of a true stem cell population, neither the agonist nor the antagonist had any effect on neurosphere size compared to control (Fig. 1B). Together, these data suggest that α1-adrenergic receptors do not directly affect activation or proliferation of adult hippocampal precursor cells.

To address whether α1-adrenergic receptors influence precursor cell differentiation, we then examined neuronal production in the neurospheres. Neurospheres generated in the presence of either cirazoline or prazosin were differentiated by growth factor removal and stained for the astrocytic marker GFAP and the immature neuronal marker βIII tubulin (Fig. 1C, D, E). All neurospheres contained GFAP-expressing astrocytes, however, only ∼40% of neurospheres contained βIII tubulin-positive neurons in the control group. As previously reported, treatment with norepinephrine resulted in a significantly higher proportion of neurospheres that expressed βIII tubulin-positive neurons (Fig. 1C, *p*<0.05). The percentage of neurospheres containing βIII tubulin-positive neurons in the cirazoline- and prazosin-treated groups was similar to that of the control group (Fig 1C).

To explore whether α1-adrenergic receptors influence adult hippocampal neurogenesis *in vivo*, we assessed proliferation, precursor cell number and number of DCX-immunopositive immature neurons. Mice expressing GFP under the Nestin promoter were used to evaluate the effects of α1-adrenergic receptor manipulation on precursor cells (Fig. 1G). Cirazoline or prazosin was administered once daily for a period of 7 days and proliferation was assessed by BrdU immunohistochemistry (Fig. 1F). Upon quantification, we found no change in the number of BrdU-positive cells in the SGZ of cirazoline- and prazosin-treated mice as compared to the vehicle-treated controls (Fig. 1I). Similarly, no change in the number of Nestin-GFP cells was observed between the control and the treatment groups (Fig. 1J), suggesting that stimulating or blocking α1-adrenergic receptors does not affect the total Nestin-positive precursor cell pool *in vivo*. Moreover, the percentage of Nestin-GFP-positive cells that were also immunopositive for GFAP (data not shown) as well as the number of newborn DCX-immunopositive neurons (Fig. 1H, K) were unchanged by α1-adrenergic receptor stimulation or blockade. Taken together these findings indicate that α1-adrenergic receptors do not appear to influence the activation, proliferation or differentiation of hippocampal precursor cells and therefore, in a 7-day treatment paradigm, do not alter adult hippocampal neurogenesis.

### Stimulation of α2-adrenergic receptors inhibits hippocampal precursor activity and decreases hippocampal neurogenesis

**Figure 2:**
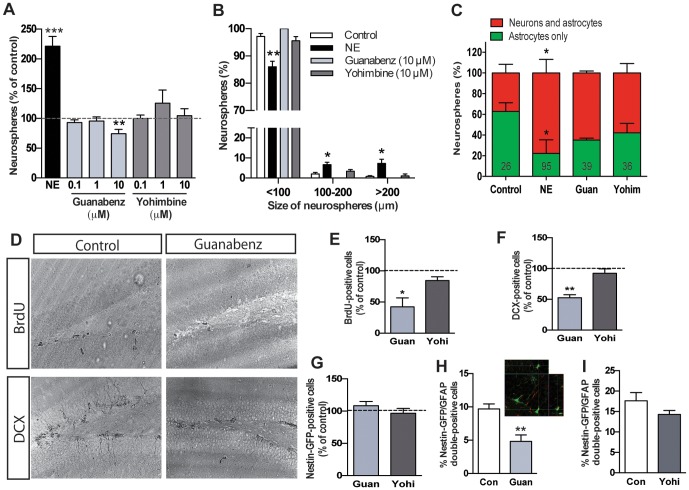
Stimulation of α2-adrenergic receptors inhibits hippocampal precursor activity and decreases neurogenesis. (A) Treatment of hippocampal precursor cells with α2-adrenergic receptor agonist at 10 uM significantly decreased neurosphere formation compared to the control. Blockade of α2-adrenergic receptors with yohimbine had no effect of neurosphere activity (n = 5 experiments). (B) Treatment with either guanabenz or yohimbine did not affect the size of hippocampal neurospheres. (C) Percentage of neurospheres displaying markers of neurons and astrocytes were similar between control, guanabenz or yohimbine treatment. As expected, a significantly larger proportion of norepinephrine-treated neurospheres contained neurons. The total number of neurospheres examined for each treatment group is indicated on the graph. (D) Representative photomicrographs of BrdU- and DCX-labeled cells from control and guanabenz treated mice. (E) Quantitative analysis revealed a significant reduction in BrdU-positive cells in SGZ of guanabenz but not yohimbine treated mice (n = 4-6 mice per group). Moreover, while neither guanabenz nor yohimbine treatment showed any changes in the Nestin-GFP-positive population (G), guanabenz (H) but not yohimbine (I) significantly reduced the percentage of Nestin-GFP/GFAP double-positive cells. Note the co-localization of GFP with GFAP in (H). The number of DCX-positive cells was also significantly reduced in guanabenz-treated mice (F). (mean±SEM, *p<0.05, **p<0.01, ***p<0.01).

Next, we examined the role of α2-adrenergic receptors in regulating precursor cell activity and hippocampal neurogenesis using a selective α2-adrenergic receptor agonist, guanabenz, and an antagonist, yohimbine. Firstly, the direct effect of guanabenz and yohimbine on adult hippocampal precursor cell population was characterized *in vitro*. Treatment with 0.1 µM and 1 µM of guanabenz had no effect, whereas 10 µM guanabenz resulted in a significant reduction in the number of neurospheres (Fig. 2A) compared to the control. This was consistent with our previous study where we had observed a similar guanabenz-evoked decline in hippocampal precursor activity in neurospheres derived from postnatal day 7 rats [15]. In addition, the neurospheres generated at this concentration were all less than 100 µm in diameter (Fig. 2B), suggestive of reduced precursor cell proliferation. Treatment of hippocampal precursor cells with the antagonist yohimbine at 100 nM, 1 µM or 10 µM did not affect the number nor the size of the neurospheres. The addition of 10 µM norepinephrine resulted in the expected ∼2-fold increase in the number of neurospheres in this assay. The percentage of neurospheres expressing both astrocytic and neuronal markers was similar in the control, 10 µM guanabenz and 10 µM yohimbine groups, suggesting that α2-adrenergic receptors do not directly regulate differentiation of hippocampal precursor cells (Fig. 2C).

To address the effects of α2-adrenergic receptors on the hippocampal precursor cell pool and immature neuron numbers in the hippocampal neurogenic niche *in vivo*, guanabenz, yohimbine or vehicle was administered systemically once daily for 7 days to Nestin-GFP mice. As reported previously [15], mice treated with guanabenz had a significantly lower percentage of BrdU-positive cells (42.3±14.4%, *p*<0.05) as compared to the vehicle-treated controls, whereas no change in the percentage of proliferating cells was observed following yohimbine treatment (84.4±6.3%, *p*>0.05; Fig. 2D, E). Intriguingly, guanabenz treatment did not alter the total number of Nestin-GFP positive cells (Fig. 2G). To further probe the population of precursor cells affected by guanabenz, we performed dual labelling for Nestin-GFP and GFAP, which has been shown to label the quiescent precursor cells, and found a significant decline in the number of double positive cells (Fig. 2H). Concomitantly, the percentage of DCX-positive immature neurons was also significantly decreased by this treatment (*p*<0.01, Fig. 2D, F) compared to the vehicle-treated control group. In contrast, yohimbine administration did not alter the total number of Nestin-GFP-positive precursors, the percentage of GFP/GFAP-positive cells (Fig. 2G, I) or DCX-positive immature neuron numbers (Fig. 2D, F) as compared to the control group.

Collectively, these findings indicate that α2-adrenergic receptors exert an inhibitory effect on hippocampal quiescent precursor cell activity that results in a decline in new neuron production in the adult hippocampus.

### Stimulation of β-adrenergic receptors activates hippocampal precursor cells and increases their proliferation

**Figure 3:**
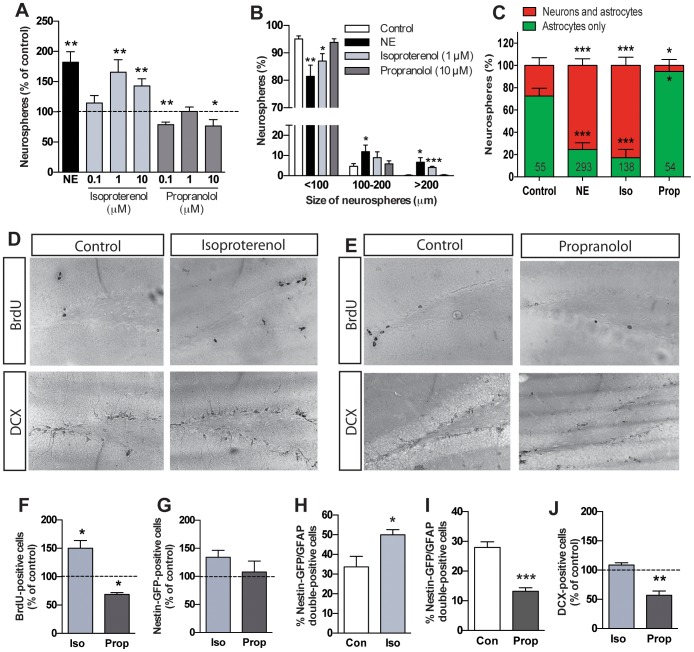
Stimulation of β-adrenergic receptors enhances hippocampal precursor activity whereas blockade inhibits precursor activity and decreases neurogenesis. (A) Treatment of primary hippocampal cells with β-adrenergic receptor agonist isoproterenol at 1 and 10 µM significantly increased where as with antagonist propranolol (0.1 and 10 µM) decreased neurosphere formation (n = 4 experiments). (B) Neurospheres obtained in the presence of isoproterenol were similar to norepinephrine-derived neurosphere, particularly the emergence of a small proportion of very large neurospheres measuring >200 µm in diameter. (C) A significantly large proportion of isoproterenol-treated neurospheres contained both neurons and astrocytes similar to that observed in norepinephrine-derived neurospheres. Proportion of neurospheres expressing neuronal marker βIII tubulin was significantly reduced in propranolol-treated neurospheres compared to control. (D, E) Representative photomicrographs of BrdU- and DCX-labeled cells from control, isoproterenol and propranolol-treated mice. (F) *In vivo* administration of isoproterenol increased whereas propranolol decreased the number of BrdU-positive cells in the SGZ (n = 4–5 mice per group). (G) While a trend towards an increase in Nestin-GFP-positive cells was obtained following isoproterenol treatment, propranolol treatment did not alter GFP-positive cell numbers in SGZ. (H) The percentage of Nestin-GFP/GFAP double-positive cells was significantly enhanced by isoproterenol treatment where as propranolol treatment (I) led to a significant reduction in double-positive cells compared to the vehicle-treated control group. (J) Propranolol treatment resulted in a significant reduction in DCX-positive immature neuron pool. (mean±SEM, *p<0.05, **p<0.01, ***p<0.01).

Finally, we examined the effects of stimulating or blocking β-adrenergic receptors using the selective β-adrenergic receptor agonist isoproterenol or the antagonist propranolol, respectively. In the neurosphere assay, treatment with 1 µM and 10 µM isoproterenol resulted in a significant, and up to ∼2-fold, increase in neurosphere number. This was consistent with our previous study, where we had found a similar increase in the number of neurospheres in presence of a selective β3-adrenergic receptor agonist BRL37344 [1]. Moreover, we found that treatment with propranolol at 0.1 µM and 10 µM led to a significant decrease in the number of neurospheres (Fig. 3A). As reported previously with norepinephrine [1], a significant increase in the size of the neurospheres was also observed in response to 1 µM isoproterenol, particularly in the number of neurospheres measuring >200 µm in diameter (Fig. 3B). While propranolol treatment did not affect the size of the neurospheres (Fig. 3B), it significantly reduced the proportion of neurospheres containing neurons (Fig. 3C).

In line with our previous findings [1], treatment of mice with isoproterenol resulted in a significant increase in the number of BrdU-positive proliferating precursor cells compared to control, whereas propranolol treatment led to a significant decline (*p*<0.05; Fig. 3D, E, F). Interestingly, treatment with isoproterenol evoked a trend toward an increase in the total number of Nestin-GFP-positive cells (*p* = 0.06; Fig. 3G), however, no change in the Nestin-GFP-positive cell number was observed following propranolol treatment. Furthermore, a significant increase in the proportion of Nestin-GFP/GFAP double-labeled cells was obtained following isoproterenol treatment as compared to the vehicle-treated control (*p*<0.01; Fig. 3H), once again reflecting an increase in the quiescent precursor cell pool and reconfirming our previous results [1]. In contrast, propranolol treatment decreased the percentage of Nestin-GFP/GFAP double-positive cells (*p*<0.001; Fig. 3I). A significant reduction in the number of DCX-positive immature neurons was also observed following propranolol treatment but the DCX-positive neuronal population was unaltered in the isoproterenol-treated mice compared to the control (Fig. 3E, J).

These results extend our previous findings and suggest that β-adrenergic receptors directly regulate both basal and stimulation-dependent activation of precursor cells. Furthermore, they also regulate the pool of DCX-positive new neurons in the adult hippocampus.

### Stimulation of α2- adrenergic receptors directly inhibits, whereas β-adrenergic receptor stimulation activates, the adult hippocampal precursor cell population

**Figure 4:**
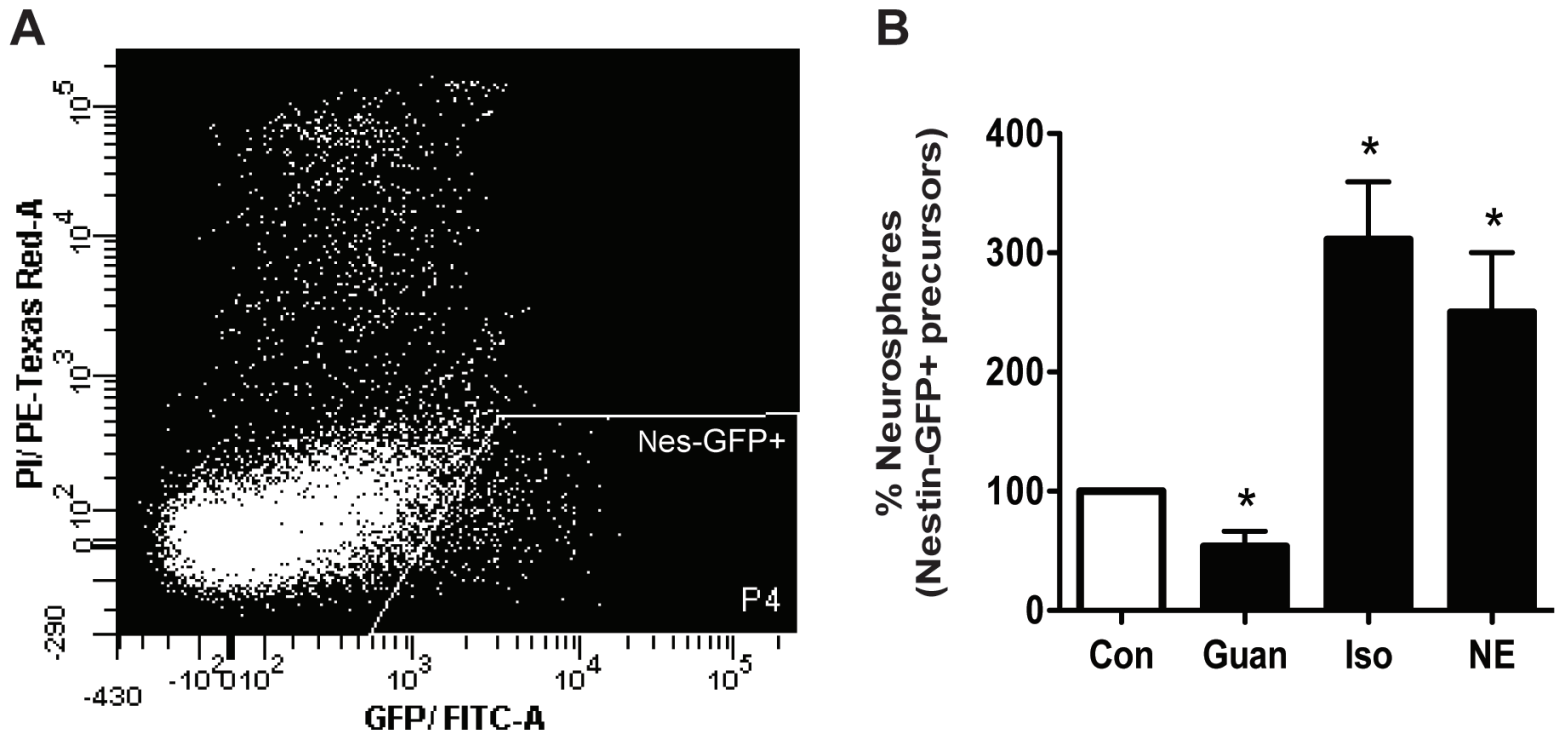
Stimulation of α2-adrenergic and β-adrenergic receptors exerts opposing effects on proliferative activity of Nestin-positive hippocampal precursor cells. (A) Nestin-positive cells from the adult hippocampus were sorted using flow cytometry based on their GFP expression. (B) Treatment with the α2-adrenergic receptor agonist guanabenz directly and significantly inhibited Nestin-GFP-positive hippocampal precursor cell proliferation as measured by a significant decline in neurosphere number. In contrast, treatment with the β-adrenergic receptor agonist isoproterenol significantly increased Nestin-GFP-positive hippocampal precursor cell proliferation with an enhanced number of neurospheres noted following isoproterenol treatment. The increased neurosphere numbers induced by isoproterenol were comparable to the induction observed following norepinephrine treatment. (mean±SEM, *p<0.05, n = 3 experiments).

The above results suggest that α2- and β-adrenergic receptors regulate the same hippocampal precursor cell pool in opposite fashions. To experimentally investigate this, we used flow cytometry to purify Nestin-GFP-positive precursor cells from the adult hippocampus (Fig. 4A). Nestin-GFP-positive cells (at a density of less than 15 cells per well) were plated in control medium or in media containing either guanabenz or isoproterenol. Treatment with medium containing norepinephrine was used as a positive control. The number of neurospheres obtained in guanabenz-treated wells was significantly reduced compared to the neurosphere number observed in control wells (*p*<0.05; n = 3; Fig. 4B). In contrast, isoproterenol treatment led to a significant (∼3-fold; *p*<0.05) increase in the number of neurospheres as compared to control conditions. This increase with isoproterenol treatment was comparable to the enhanced neurosphere number observed in the presence of norepinephrine. These results provide strong evidence that α2- and β-adrenergic receptors directly regulate the proliferative activity of the Nestin-positive precursor cell population in an opposing fashion.

## Discussion

Our *in vitro* neurosphere assay results, as well as the *in vivo* findings from administration of adrenergic receptor-selective agonists and antagonists, demonstrate that α2- and β-adrenergic receptors exert direct and opposing effects on the regulation of quiescent neural precursor cell activity, whereas α1-adrenergic receptors do not appear to have a significant role in the regulation of adult hippocampal neurogenesis (see Table 2).

**Table 2:** Summary of the stage-specific effects of sub-class selective adrenergic receptor agonists and antagonists on hippocampal precursor activity and neurogenesis.

AR Receptor pharmacology	Neurospheres	Proliferation (BrdU^+^ cells)	Nestin-GFP^+^ cells	Nes-GFP/GFAP cells	Immature Neurons (DCX^+^)
Cirazoline	NC	NC	NC	NC	NC
Prazosin	NC	NC	NC	NC	NC
Guanabenz	←	←	NC	←	←
Yohimbine	NC	NC	NC	NC	NC
Isoproterenol	→	→	→^*^	→	NC
Propranolol	←	←	NC	←	←

NC: No change;

→: Increased;

←: Decreased;

→^*^: *p* = 0.06.

The demonstration that guanabenz significantly decreased the number of neurospheres, as well as the number of BrdU-positive cells in the SGZ, suggests that α2-adrenergic receptors expressed by hippocampal precursors play an inhibitory role in regulating the cell cycle. Interestingly, we did not observe any effect on precursor cell turnover following α2-adrenergic receptor blockade. These results suggest that α2-adrenergic receptor exert a stimulation-induced, but not a basal tonic, inhibitory effect on adult hippocampal precursor cell turnover. On the other hand, β-adrenergic receptors play an important role in regulating the proliferation of precursor cells under basal condition. Importantly, a direct effect on precursor cell activation and proliferation observed following stimulation α2- and β-adrenergic receptors on purified Nestin-GFP^+^ precursor cells is in agreement with our previous data showing expression of these receptor subtypes on freshly isolated precursors and in neurospheres [1], [15]. This is further supported by our previous finding that a decrease in proliferation persists following α2-adrenergic receptor stimulation in dopamine-β-hydroxylase knock-out mice, suggesting that the effects are mediated by mechanisms involving post-synaptic heteroceptors rather than pre-synaptic autoreceptors [15]. While we have not directly examined whether α2- or β-adrenergic receptors regulate precursor cell survival, no change in the number of Nestin-GFP-positive cells following guanabenz or propranolol treatment (Table S1) suggest that a decrease in precursor cell proliferation rather than their death leads to an overall reduction in BrdU and neurosphere numbers.

A major novel finding of our study is that the opposing effects of α2- and β- adrenergic receptors on cell turnover are likely to involve the same class of hippocampal precursors, those expressing Nestin. Nestin-GFP/GFAP-expressing cells in the SGZ have been proposed to contain quiescent precursor cell population (reviewed in [5]). The finding that stimulation of α2-adrenergic receptors reduced the percentage of Nestin-GFP/GFAP double-positive cells and directly inhibited the ability of Nestin-positive precursor cells to respond to mitogens in the neurosphere assay, whereas stimulation of β-adrenergic receptors increased both the percentage of Nestin-GFP/GFAP double-positive cells *in vivo* and significantly increased the number of neurospheres *in vitro*, strongly suggests that these noradrenergic receptors maintain the proliferative balance between inhibition and stimulation of these Nestin-positive hippocampal precursor cells. Moreover, as neurosphere number is considered to be a read-out of the ‘active’ precursor population capable of proliferation in the presence of growth factors, we speculate that the changes observed in the percentage of Nestin-GFP/GFAP double-positive cells within the neurogenic niche may in fact reflect the activation status of quiescent precursor populations. Further support for this idea comes from our evidence that treatment with the β ˜adrenergic receptor antagonist propranolol led to a significant decrease in neurosphere numbers, which was paralleled with a significant decline in the percentage of Nestin-GFP/GFAP double-positive precursors within the SGZ. Our previous studies suggest that the norepinephrine-responsive population is likely to be distinct from the population of precursors that responds to neuronal activity in the adult hippocampal neurogenic niche [2], [5]. Although our data from flow cytometry-based experiments show that the opposing effects of α2- and β-adrenergic receptors are indeed mediated on the same class of Nestin-GFP-expressing hippocampal precursors, further molecular evidence of possible subclasses present within the Nestin-GFP/GFAP double-positive precursor pool cannot yet be ruled out. However, it does indicate that stimulation of β-adrenergic receptor with isoproterenol directly activates an otherwise quiescent Nestin-GFP-positive precursor cells that now become responsive to mitogens (EGF+bFGF).

Nonetheless, an important question that arises from this study is the nature of α2- and β-adrenergic receptor balance within the quiescent neural precursor population following norepinephrine release in the hippocampus, which ultimately regulates precursor cell proliferation and neurogenesis.

A possible answer lies in the effector systems downstream of α2- and β-adrenergic receptors. Whereas α2-adrenergic receptors are coupled to inhibitory G (Gi) proteins, resulting in inhibition of adenylate cyclase, β-adrenergic receptors activate adenylate cyclase through stimulatory G (Gs) proteins, thereby increasing the levels of cAMP [14], [27], [28]. Previous studies have reported that activation of the cAMP and cAMP response element-binding protein (CREB) pathway as a positive regulator of hippocampal neurogenesis and have suggested its role in mediating the neurogenic effects of neurotransmitters and antidepressants [29]–[34]. Specifically, the study by Nakagawa and colleagues [34] suggested an enhancement in CREB phosphorylation as a possible mechanism underlying precursor cell proliferation. Therefore, it is possible that Gs versus Gi protein coupling of these adrenergic receptors leads to changes in the levels of cAMP and phospho-CREB, which may dictate the final outcome of stimulation versus inhibition of neural precursor cell proliferation. Furthermore, the significant reduction in the DCX-positive immature neuron population observed following stimulation of α2- or inhibition of β-adrenergic receptors could be attributed to downregulation of the cAMP-CREB pathway, which has also previously been shown to regulate hippocampal precursor cell differentiation [34]. Given that the hippocampal precursor cells express both α2- and β-adrenergic receptors, however, differences in stoichiometry of these receptors, receptor coupling and oligomerization states as well as possible heterocomplex formation could also serve to play an important role in defining the nature of effects evoked by norepinephrine on proliferation and differentiation of these precursor cells.

A puzzling finding of this study was that isoproterenol treatment resulted in enhanced proliferation both *in vivo* and *in vitro*, but only increased neuronal production *in vitro*. This suggests that while stimulation of β-adrenergic receptors leads to precursor cell activation and proliferation *in vivo*, additional growth factors such as those present in the neurosphere assay may be required to increase neuronal differentiation, thus de-coupling activation/proliferation of hippocampal precursors from their differentiation. One cannot preclude the possibility that sustained isoproterenol treatment may be required for differentiation or that the effects on neuronal differentiation may only emerge in a delayed fashion (more than two weeks) following the activation of quiescent precursor cells. Future studies are required to address whether specific adrenergic receptors can influence nestin-GFP-positive precursors to adopt astrocytic and oligodendroglial fates.

To date, the role of α1-adrenergic receptors in regulating various stages of neurogenesis in the hippocampus has not been characterized in detail. Earlier studies have reported a positive role for α1-adrenergic receptors in regulating neurogenesis in the subventricular zone and subgranular zone using either transgenic mice overexpressing α1-adrenergic receptors or selective agonists [26], [35]. These studies also demonstrated improvement in learning and memory following long-term (up to 9 months) treatment with the α1-adrenergic receptor agonist cirazoline, however, they did not report any influence on hippocampal neurogenesis. Given that we found no change in either precursor cell activation/proliferation nor in immature neuronal cell number in the dentate gyrus following stimulation or inhibition of α1-adrenergic receptor activity, it is possible that the beneficial effects of α1-adrenergic receptors in improving cognition and mood involve mechanisms independent of neurogenesis. Alternatively, a treatment regime of more than 7 days may be required to exert positive effects on adult hippocampal neurogenesis.

Finally, the differential effect on the proliferative activity of hippocampal precursor cells exerted by α2- and β-adrenergic receptors is particularly interesting in the context of the effects of stress [36] and antidepressant treatments. Chronic stress models that result in hippocampal neurogenic decline have been reported to be associated with enhanced α2-adrenergic receptor expression and binding within limbic brain regions [37], [38]. In contrast, chronic treatments with antidepressants that exert powerful pro-neurogenic effects have been linked to a reduction in α2-adrenergic receptor expression and signaling [39]–[41]. These results suggest a model in which environmental influences such as stress, aging and physical exercise may serve to influence the extent of α2- versus β-adrenergic receptor expression or signaling within the quiescent precursor cell populations, thereby differentially modulating the effects of norepinephrine on precursor cell activation and proliferation. This has clinical relevance given our previous findings that combination therapy with drugs that block α2-adrenergic receptor activity concomitant with elevation in the levels of norepinephrine via transporter blockade (norepinephrine re-uptake inhibitors) accelerates both neurogenic and antidepressant-like effects [15]. Interestingly, a randomized double-blind study in humans has reported a hastened antidepressant response in clinically depressed subjects treated with a combination of fluoxetine (selective serotonin re-uptake inhibitor) and yohimbine [42].

Overall, our results demonstrate that stimulation of α2-adrenergic receptors directly inhibit whereas that of β-adrenergic receptors enhance Nestin-positive precursor cell activation and proliferation, and implicate the balance from inhibitory to pro-stimulatory effect as a possible novel mechanism to speed up neurogenic effects of norepinephrine-elevating drugs.

## Supporting Information

Table S1 **Quantification showing average number of BrdU-, DCX- and Nestin-GFP-labeled cells in mice treated with selective adrenergic receptor agonists and antagonists.**
(DOCX)
