# Supplementary material for: Opposing Effects of α2- and β-Adrenergic Receptor Stimulation on Quiescent Neural Precursor Cell Activity and Adult Hippocampal Neurogenesis
Source: PLoS One. 2014 Jun 12;9(6):e98736. doi: 10.1371/journal.pone.0098736 (PMC4055446; doi:10.1371/journal.pone.0098736)
Supplement: Table S1 — Quantification showing average number of BrdU-, DCX- and Nestin-GFP-labeled cells in mice treated with selective adrenergic receptor agonists and antagonists. (DOCX) [file pone.0098736.s001.docx]

**Table S1: Quantification showing average number of BrdU-, DCX- and Nestin-GFP-labeled cells in mice treated with selective adrenergic receptor agonists and antagonists.**

| **Adrenergic Receptor** | **Treatment** | **Cells per section** | | |
| --- | --- | --- | --- | --- |
|  |  | **BrdU** | **DCX** | **Nestin-GFP** |
| **α1** | **Control** | 78.25 ± 9.877 | 95.87 ± 11.886 | 133 ± 9.668 |
|  | **Cirazoline** | 78.75 ± 6.474 | 69.83 ± 9.837 | 129 ± 14.926 |
|  | **Control** | 84.7 ± 7.289 | 42.05 ± 2.992 | 117.16 ± 5.6 |
|  | **Prazosine** | 67.87 ± 11.446 | 40.68 ± 8.035 | 101.7 ± 15.087 |
| **α2** | **Control** | 83.16 ± 14.96 | 95.87 ± 11.886 | 133 ± 9.668 |
|  | **Guanabenz** | 35.19 ± 12.021***** | 50.4 ± 4.76* | 149.62 ± 8.442 |
|  | **Control** | 56.7 ± 7.239 | 51.64 ± 6.398 | 111.81 ± 4.951 |
|  | **Yohimbine** | 47.85 ± 3.561 | 47.65 ± 3.707 | 108.06 ± 8.384 |
| **β** | **Control** | 60.68 ± 5.418 | 86.1 ± 4.594 | 80.31 ± 5.185 |
|  | **Isoproterenol** | 96.14 ± 6.929* | 93.615 ± 3.338 | 107.56 ± 9.956 |
|  | **Control** | 96.42 ± 9.989 | 66.53 ± 4.901 | 122.5 ± 20.467 |
|  | **Propranolol** | 66.35 ± 3.082* | 37.92 ± 4.842* | 132 ± 23.76 |
